# Association Between Periodontitis Severity and Oral Health-Related Quality of Life: A Cross-Sectional Study Using the 2017 Periodontitis Classification

**DOI:** 10.3390/healthcare14172678

**Published:** 2026-08-23

**Authors:** Gabriela Zambrano Manzaba, Jazmin Rodríguez Grajales, Hector Alfredo Lema Gutierrez, Luis Chauca Bajaña, Mónica Gabriela Magaña Pérez, Gema Nallely Mendoza Manzaba, Rito Alfonso Salazar Roman, Daniela Galicia-Diez Barroso, Luis David Abeijon Malvaez

**Affiliations:** 1 Faculty of Dentistry, Universidad Católica de Santiago de Guayaquil (UCSG), Guayaquil 090101, Ecuador; 2 Faculty of Dentistry, Universidad Tecnológica de México (UNITEC), Mexico City 11320, Mexico; 3 Postgraduate Program in Periodontology, Faculty of Dentistry, Universidad Tecnológica de México (UNITEC), Mexico City 11320, Mexico; monica_magana@my.unitec.edu.mx; 4 School of Medicine, Faculty of Health Sciences, Universidad Católica de Santiago de Guayaquil (UCSG), Guayaquil 090615, Ecuador; 5 Department of Oral Public Health, Division of Graduate Studies and Research, Faculty of Dentistry, National Autonomous University of Mexico (UNAM), Mexico City 01020, Mexico; 6 Mexican Institute for Health and Welfare (IMSS-BIENESTAR), Mexico City 01020, Mexico; luis.abeijon@imssbienestar.gob.mx

**Keywords:** periodontitis, oral health-related quality of life, OHIP-14, disease severity

## Abstract

Background: Periodontitis is a chronic inflammatory disease characterized by progressive destruction of the tooth-supporting tissues and may substantially impair oral health-related quality of life (OHRQoL). Although the clinical consequences of periodontitis are well documented, the extent to which disease severity is associated with patient-reported outcomes remains insufficiently understood. This study aimed to evaluate the association between periodontitis severity and OHRQoL and to examine sociodemographic, behavioral, and systemic factors associated with poorer OHRQoL. Methods: A cross-sectional analytical study was conducted among 136 adult patients diagnosed with stage II or stage IV periodontitis at the Periodontology Clinic of Universidad Tecnológica de México (UNITEC). OHRQoL was assessed using the Oral Health Impact Profile for Periodontal Disease (OHIP-14-PD). Sociodemographic characteristics, smoking status, and diabetes mellitus were recorded using a structured questionnaire. Periodontal status was determined through standardized clinical and radiographic examinations. The internal consistency of the OHIP-14-PD was evaluated using Cronbach’s alpha coefficient. Bivariate analyses were performed using non-parametric tests. An exploratory, partially adjusted multivariable linear regression model including sex, marital status, diabetes mellitus, and periodontitis stage was also performed. Results: Stage IV periodontitis was more prevalent than stage II periodontitis (55% vs. 45%). The mean OHIP-14-PD score was 24.9 ± 13.7, and the instrument demonstrated excellent internal consistency (Cronbach’s α = 0.89). Patients with stage IV periodontitis had significantly higher OHIP-14-PD scores than those with stage II disease (30.1 ± 12.4 vs. 18.6 ± 12.6; *p* < 0.001), indicating poorer OHRQoL. In the bivariate analyses, poorer OHRQoL was also observed among current smokers, participants with diabetes mellitus, those with lower educational attainment, and those with lower socioeconomic status (*p* < 0.05). In the exploratory partially adjusted model, male sex (β = 4.2; 95% CI: 0.1–8.2; *p* = 0.042), diabetes mellitus (β = 6.5; 95% CI: 2.1–10.9; *p* = 0.004), and stage IV periodontitis (β = 4.6; 95% CI: 0.4–8.7; *p* = 0.031) were associated with higher OHIP-14-PD scores. Conclusions: In this single-center cross-sectional sample, patients with stage IV periodontitis reported higher OHIP-14-PD scores than those with stage II periodontitis. These findings indicate an association within the studied clinical population and should not be interpreted as evidence of a causal effect or as directly generalizable to broader populations. Larger multicenter longitudinal studies including patients across all periodontitis stages are required to confirm the magnitude and external validity of this association.

## 1. Introduction

Periodontitis is a chronic, multifactorial inflammatory disease associated with dysbiotic dental biofilm and characterized by progressive destruction of the tooth-supporting tissues, including clinical attachment loss, periodontal pocket formation, radiographic bone loss, and, in advanced cases, tooth loss [1,2,3]. Beyond its clinical consequences, periodontitis may impair essential oral functions such as mastication, speech, aesthetics, and social interaction, thereby affecting patients’ perceived well-being and daily activities [4,5].

The 2017 World Workshop introduced a staging and grading system that classifies periodontitis according to severity, complexity, extent, distribution, and risk of progression [6]. In this framework, stage II periodontitis represents a moderate form of disease, whereas stage IV periodontitis reflects advanced periodontal destruction, often accompanied by tooth loss, masticatory dysfunction, occlusal instability, and the need for complex rehabilitation [7]. Therefore, evaluating the impact of different periodontal stages on patient-centered outcomes is clinically relevant.

OHRQoL provides a broader understanding of how oral diseases affect physical, psychological, and social dimensions of health [8]. The Oral Health Impact Profile (OHIP) and its short version, OHIP-14, are among the most widely used instruments for assessing the subjective impact of oral conditions [9,10]. Previous systematic reviews and clinical studies have shown that periodontal disease negatively affects OHRQoL, with greater impairment generally observed in patients with more severe forms of periodontitis [11,12,13,14].

Recent evidence also suggests that periodontitis staging and grading are associated with poorer OHRQoL, supporting the clinical value of the 2017 classification beyond diagnostic purposes [15,16]. In addition, systemic and behavioral factors such as diabetes mellitus and smoking may contribute to both periodontal disease severity and patient-perceived oral health burden [17,18]. Diabetes has a well-established bidirectional relationship with periodontitis, while smoking has been consistently associated with increased risk and progression of periodontal destruction [19,20].

Despite growing evidence of an association between periodontitis and impaired OHRQoL, limited evidence has examined whether clinically distinct levels of disease severity defined according to the 2017 periodontitis classification are associated with different patient-perceived burdens. In particular, direct comparisons between stage II periodontitis, representing moderate periodontal destruction, and stage IV periodontitis, characterized by advanced destruction, functional impairment, and greater treatment complexity, remain limited. The present study addresses this gap by integrating the 2017 staging framework with the periodontal disease-specific OHIP-14-PD instrument in a Mexican clinical population. Therefore, this study aimed to compare OHRQoL between patients with stage II and stage IV periodontitis and to explore the sociodemographic, behavioral, and systemic factors associated with poorer OHRQoL.

## 2. Materials and Methods

### 2.1. Study Design and Ethical Considerations

A cross-sectional analytical study was conducted among adult patients attending the Periodontology Clinic of the Faculty of Dentistry at Universidad Tecnológica de México (UNITEC) from 15 January 2025 to 31 January 2026. The study aimed to evaluate the association between periodontitis severity and OHRQoL. The research protocol was reviewed and approved by the Research and Bioethics Committee of UNITEC (approval number: 2022-035 V2) and was conducted in accordance with the ethical principles of the Declaration of Helsinki for research involving human subjects. Prior to enrollment, all participants received detailed information regarding the objectives and procedures of the study and provided written informed consent. Participation was voluntary, and participants were informed of their right to withdraw from the study at any stage without any academic or clinical consequences. Eligible participants were adults aged 18 years or older with a confirmed diagnosis of stage II or stage IV periodontitis based on comprehensive clinical and radiographic periodontal examination. Patients with incomplete clinical records, missing questionnaire data, systemic conditions compromising periodontal assessment, or refusal to participate were excluded from the study. Data collection was performed using a standardized protocol that included a comprehensive periodontal examination and administration of a validated questionnaire. Sociodemographic and behavioral information was obtained through structured face-to-face interviews conducted by trained researchers, whereas periodontal clinical parameters were recorded under standardized clinical conditions by calibrated examiners. To ensure data quality, participants with complete periodontal assessments and OHIP-14-PD outcome data were included in the descriptive and bivariate analyses. Participants with missing information for one or more covariates required for the multivariable model were excluded only from the complete-case regression analysis. The study was reported according to the Strengthening the Reporting of Observational Studies in Epidemiology (STROBE) guidelines.

### 2.2. Participants and Sample Size Calculation

The study population consisted of adult patients attending the Periodontology Clinic of the Faculty of Dentistry at Universidad Tecnológica de México (UNITEC). Participant recruitment and data collection were conducted from 15 January 2025 to 31 January 2026. A non-probability consecutive sampling strategy was used, whereby all patients attending the clinic during the recruitment period were sequentially assessed for eligibility. Participation was voluntary, and participants were informed that they could withdraw from the study at any time without affecting their current or future clinical care.

Eligible participants were adults aged 18 years or older with a confirmed diagnosis of stage II or stage IV periodontitis based on comprehensive clinical and radiographic periodontal examinations. Patients with incomplete clinical records, missing OHIP-14-PD outcome data, systemic conditions that could compromise periodontal assessment, or refusal to participate were excluded from the study.

A total of 154 patients were assessed for eligibility. Of these, 10 were excluded because they did not meet the eligibility criteria: seven were diagnosed with stage I or stage III periodontitis, two presented systemic conditions that could compromise periodontal assessment, and one had incomplete clinical records. Among the 144 eligible patients invited to participate, three declined participation and five did not complete the periodontal examination or the OHIP-14-PD questionnaire. Ultimately, 136 participants were enrolled and included in the descriptive and bivariate analyses, corresponding to a participation rate of 94.4%.

The sample size was calculated using OpenEpi software, version 3.01. The estimation was based on the comparison of mean OHIP-14-PD scores between two independent groups: patients with stage II periodontitis, considered the reference group, and patients with stage IV periodontitis, considered the exposed group. A moderate expected effect size of Cohen’s d = 0.50 was assumed, with a two-sided confidence level of 95% and statistical power of 80%. Under these assumptions, the minimum required sample size was 64 participants per group, corresponding to 128 participants in total. To compensate for potential incomplete questionnaires or missing clinical data, an additional 5% was added, resulting in a target sample size of approximately 134 participants. The final sample included 136 participants, exceeding the minimum required sample size. This sample-size calculation was specifically intended to provide adequate statistical power for the primary comparison of OHIP-14-PD scores between patients with stage II and stage IV periodontitis. However, meeting the prespecified sample-size requirement does not eliminate the limitations associated with single-center recruitment and does not support population-level estimates, causal inference, subgroup analyses, or broad external generalization.

### 2.3. Sociodemographic and Clinical Assessment

Data collection was performed using a structured and standardized questionnaire designed to obtain sociodemographic, behavioral, systemic, and clinical information from all participants. The variables collected included age (years), sex (male/female), marital status (single, married, divorced, or widowed), educational level (primary, secondary, or higher education), and socioeconomic status. Smoking status was recorded as a dichotomous variable (yes/no) based on participants’ self-reported current smoking habits through the question, “Do you currently smoke?” Diabetes mellitus was similarly recorded as a binary variable (yes/no) based on a previous medical diagnosis self-reported by the participant. OHRQoL was assessed using the Oral Health Impact Profile for Periodontal Disease (OHIP-14-PD), a periodontal disease-specific adaptation of the original OHIP and its abbreviated 14-item version [7,8]. The questionnaire comprises 14 items covering seven conceptual domains: functional limitation, physical pain, psychological discomfort, physical disability, psychological disability, social disability, and handicap. Each item was scored using a five-point Likert scale: 0 = never, 1 = hardly ever, 2 = occasionally, 3 = frequently, and 4 = very often. Item scores were summed without weighting to obtain a total score ranging from 0 to 56, with higher scores indicating a greater negative impact on OHRQoL. No fixed retrospective recall period was specified; therefore, participants responded according to the perceived frequency of oral health impacts at the time of questionnaire administration. The internal consistency of the OHIP-14-PD in the present sample was evaluated using Cronbach’s alpha coefficient and demonstrated excellent reliability (Cronbach’s α = 0.89).

### 2.4. Periodontal Assessment

Periodontal status was evaluated through comprehensive clinical and radiographic examinations performed under standardized conditions. Clinical periodontal parameters included probing depth (PD) and clinical attachment loss (CAL), which were measured in millimeters at periodontal sites using a calibrated North Carolina periodontal probe. All clinical findings were systematically recorded in a structured periodontal chart.

Radiographic assessment was conducted to evaluate alveolar bone loss and to support the periodontal diagnosis and staging process. The diagnosis and classification of periodontitis were established according to the 2017 World Workshop classification of periodontal and peri-implant diseases and conditions proposed by Papapanou and Tonetti [2,21].

For the purposes of this study, only patients diagnosed with stage II or stage IV periodontitis were included. These two stages were intentionally selected to represent clinically distinct levels of periodontal severity, functional burden, and treatment complexity, thereby allowing a direct comparison of OHRQoL between moderate and advanced disease. Stage II was considered the reference category because it represents established periodontal destruction without tooth loss attributable to periodontitis or major functional impairment, whereas stage IV reflects advanced disease frequently accompanied by tooth loss, masticatory dysfunction, occlusal instability, and complex rehabilitation needs. Stage I and stage III were not included because the study was not designed to evaluate the entire severity continuum or a dose–response relationship across all periodontal stages; rather, it focused on maximizing the clinical contrast between moderate disease and advanced, functionally compromised periodontitis. Accordingly, the findings should be interpreted as a comparison between these two predefined clinical categories and not extrapolated as evidence of a progressive gradient across all four stages. Stage II periodontitis was defined by the presence of clinical attachment loss of 3–4 mm and radiographic bone loss extending to 15–33% of the root length, without tooth loss attributable to periodontitis. In contrast, stage IV periodontitis was characterized by severe periodontal destruction, extensive clinical attachment loss, advanced alveolar bone loss, and tooth loss related to periodontitis, frequently associated with functional impairment such as masticatory dysfunction, occlusal instability, or the need for complex oral rehabilitation.

To ensure consistency and reproducibility of the measurements, all periodontal examinations were performed according to standardized clinical protocols by previously trained and calibrated examiners. Prior to data collection, examiner calibration was performed in non-study patients to assess measurement reproducibility. Intra-examiner reliability for probing depth and clinical attachment loss measurements demonstrated an intraclass correlation coefficient (ICC) greater than 0.85, indicating excellent agreement.

### 2.5. Statistical Analysis

Data were entered into a database using EpiData software (version 3.1; EpiData Association, Odense, Denmark) and subsequently analyzed using Stata statistical software (version 14.2; StataCorp LLC, College Station, TX, USA). Continuous variables, including age and OHIP-14-PD scores, were summarized using means and standard deviations or medians and interquartile ranges, according to their distribution. Categorical variables were described using absolute and relative frequencies. The distribution of OHIP-14-PD scores was evaluated using the Shapiro–Wilk normality test. Because the outcome variable showed a non-normal distribution, non-parametric statistical methods were used for the bivariate analyses. Comparisons between two independent groups were performed using the Mann–Whitney U test, whereas comparisons involving more than two groups were assessed using the Kruskal–Wallis test.

An exploratory multivariable linear regression model was constructed using the total OHIP-14-PD score as the dependent variable. Covariate selection was based on clinical relevance, conceptual domain representation, and model parsimony rather than exclusively on statistical significance in the bivariate analyses. Sex was retained as a core demographic variable and potential confounding factor, irrespective of its non-significant bivariate association with OHIP-14-PD scores. Marital status, diabetes mellitus, and periodontitis stage were included to represent social, systemic, and clinical domains, respectively. Given the analytical sample of 128 complete cases and the number of parameters required by categorical sociodemographic variables with multiple levels, the model was intentionally restricted to reduce the risk of overparameterization and unstable coefficient estimates. Age, smoking status, educational level, and socioeconomic status were not included in this exploratory model. Educational level and socioeconomic status were considered conceptually related indicators of social conditions, and their simultaneous inclusion would have further increased the number of model parameters. Therefore, the analysis should be interpreted as exploratory and partially adjusted rather than as a fully adjusted etiological model. The exclusion of these potentially relevant variables may have resulted in residual confounding, and the reported coefficients should not be interpreted as independent or causal effects.

Sex, diabetes mellitus status, periodontitis stage, and OHIP-14-PD scores were complete for all 136 participants, whereas marital status was missing for eight participants (5.9%). The multivariable regression was conducted using a complete-case approach, resulting in an analytical sample of 128 participants. No missing-data imputation was performed. The assumptions of linearity, homoscedasticity, independence, and normality of residuals were evaluated. Multicollinearity among the independent variables was assessed using the variance inflation factor (VIF), and the explanatory capacity of the model was evaluated using the coefficient of determination (R^2^). Regression coefficients (β), their corresponding 95% confidence intervals (95% CI), and *p*-values were reported. All statistical tests were two-tailed, and a *p*-value < 0.05 was considered statistically significant.

## 3. Results

### 3.1. Sociodemographic Characteristics and Risk Factors

A total of 136 patients were included in the study. The mean age was 53.0 ± 12.8 years, and 60% (*n* = 82) were female. Regarding marital status, 39% (*n* = 51) were married. In terms of educational level, 31% (*n* = 45) had completed secondary education, and 67.9% (*n* = 91) were classified as having a high socioeconomic status according to the AMAI index.

With respect to risk factors, 43% (*n* = 58) of participants reported current smoking, and 51% (*n* = 68) had a previous diagnosis of type 2 diabetes mellitus.

### 3.2. Periodontal Status and Oral Health-Related Quality of Life

Regarding periodontal status, 45% (*n* = 61) of participants were diagnosed with stage II periodontitis, while 55% (*n* = 75) presented stage IV disease.

The mean overall OHIP-14-PD score was 24.9 ± 13.7, with observed scores ranging from 0 to 56. Higher OHIP-14-PD scores indicate a greater negative impact of oral health conditions on patients’ OHRQoL. The OHIP-14-PD instrument demonstrated excellent internal consistency in the present sample, with a Cronbach’s alpha coefficient of 0.89.

### 3.3. Bivariate Analysis

Higher OHRQoL scores, indicating poorer OHRQoL, were observed among participants with lower educational attainment, lower socioeconomic status, smoking habit, diabetes mellitus, and more severe periodontal disease (Table 1).

No statistically significant differences in OHRQoL scores were identified according to sex (*p* = 0.326). However, significant differences were observed according to marital status (*p* < 0.001), with widowed participants demonstrating the highest OHRQoL scores, whereas divorced individuals presented the lowest scores.

Participants with primary educational attainment demonstrated significantly poorer OHRQoL compared with those with higher education (*p* < 0.001). Likewise, individuals with low socioeconomic status exhibited significantly higher OHRQoL scores than participants with high socioeconomic status (*p* < 0.001).

Current smokers demonstrated significantly poorer OHRQoL compared with non-smokers (29.7 ± 12.9 vs. 21.2 ± 13.1; *p* = 0.001). Similarly, participants with diabetes mellitus reported significantly worse OHRQoL scores than non-diabetic individuals (29.0 ± 12.9 vs. 20.6 ± 13.1; *p* < 0.001).

Regarding periodontal severity, patients diagnosed with stage IV periodontitis exhibited significantly poorer OHRQoL compared with those presenting stage II disease (30.1 ± 12.4 vs. 18.6 ± 12.6; *p* < 0.001). The difference in OHIP-14-PD scores between stage IV and stage II periodontitis was additionally quantified using a non-parametric effect-size estimate with its corresponding 95% confidence interval (Table 1).

### 3.4. Multivariable Analysis

Of the 136 participants included in the descriptive and bivariate analyses, 128 had complete information for all variables included in the multivariable model. Eight participants were excluded from the complete-case regression analysis because of missing data for one or more model covariates. The model assumptions were adequately met. The residuals did not significantly deviate from normality (*p* = 0.4276), their mean was not significantly different from zero (*p* = 0.99), and no evidence of problematic multicollinearity was identified (VIF = 2.02). The exploratory partially adjusted model explained 38% of the variability in OHIP-14-PD scores (R^2^ = 0.38).

In the exploratory partially adjusted multivariable model, which included sex, marital status, diabetes mellitus, and periodontitis stage, male participants had higher OHIP-14-PD scores than female participants (β = 4.2; 95% CI: 0.1–8.2; *p* = 0.042). Participants with diabetes mellitus also had higher OHIP-14-PD scores than those without diabetes mellitus (β = 6.5; 95% CI: 2.1–10.9; *p* = 0.004). Divorced participants had lower OHIP-14-PD scores than single participants (β = −10.9; 95% CI: −17.7 to −4.2; *p* = 0.002). Given the observational design and the incomplete adjustment for relevant socioeconomic, behavioral, and demographic factors, this finding should not be interpreted as evidence of a protective effect of divorced marital status. Finally, stage IV periodontitis was associated with higher OHIP-14-PD scores compared with stage II periodontitis (β = 4.6; 95% CI: 0.4–8.7; *p* = 0.031) (Table 2). Because age, smoking status, educational level, and socioeconomic status were not included in the model, all reported coefficients should be interpreted as exploratory, partially adjusted associations rather than as independent or causal effects.

## 4. Discussion

The present study identified a significant association between periodontitis severity and OHRQoL, with higher OHIP-14-PD scores observed among patients with stage IV periodontitis than among those with stage II disease. This finding suggests that advanced periodontitis may be accompanied by a greater patient-perceived oral health burden. However, because of the cross-sectional design and the partially adjusted multivariable model, the results should not be interpreted as demonstrating that periodontitis severity directly caused poorer OHRQoL or as establishing periodontal severity as an independent determinant of quality of life. The observed association may also have been influenced by residual confounding, reverse causality, other oral conditions, and unmeasured psychosocial or behavioral factors.

Contemporary scientific evidence has consistently demonstrated that the impact of periodontitis on OHRQoL progressively increases as periodontal destruction advances [22,23]. In this regard, the present findings are biologically and clinically coherent, considering that patients with stage IV periodontitis commonly present tooth mobility, loss of posterior support, esthetic impairment, functional alterations, and the need for complex rehabilitation. These conditions not only compromise masticatory capacity and oral function but also affect psychological and social dimensions, ultimately modifying individuals’ perceptions of well-being and health.

Several clinically plausible factors may contribute to the association between advanced periodontitis and poorer OHRQoL; however, these mechanisms were not directly assessed in the present study. Severe periodontal destruction compromises occlusal stability and reduces masticatory efficiency, limiting food selection and impairing essential functions such as mastication and speech. Furthermore, tooth mobility and esthetic alterations may generate social insecurity, emotional discomfort, and reduced interpersonal interaction. Uy et al. [24] demonstrated that loss of posterior support and impaired masticatory function are closely associated with more advanced stages of periodontitis and poorer quality of life. Complementarily, recent studies have reported that severe periodontal destruction significantly affects daily oral functioning and psychosocial perception, particularly among older adults [25]. Collectively, these findings support the hypothesis that periodontal severity exerts a cumulative effect on the subjective perception of oral disability.

The findings of the present study also support the clinical utility of the staging and grading classification proposed by the 2017 World Workshop. Beyond its diagnostic value, the contemporary classification appears to adequately reflect the functional and psychosocial burden associated with advanced periodontal disease [15,16,26]. In this context, worsening periodontal stage and grade have been shown to affect not only perceived oral health but also dimensions related to sleep quality, general health perception, and overall well-being [27]. These findings reinforce the need to incorporate patient-centered outcomes into routine periodontal assessment, particularly in individuals with advanced disease.

Diabetes mellitus was associated with higher OHIP-14-PD scores in the partially adjusted model. Although inflammatory and metabolic interactions between diabetes and periodontitis provide biological plausibility for this finding, glycemic control, diabetes duration, medication use, and biochemical markers were not assessed. Moreover, diabetes status was self-reported. Consequently, the present study cannot determine the mechanisms underlying this association, and the result should be interpreted cautiously because misclassification and residual confounding cannot be excluded. Oliveira et al. [28] reported that patients with chronic systemic diseases and periodontitis exhibit significant deterioration in OHRQoL, emphasizing the cumulative effect of systemic and oral conditions on overall well-being. Likewise, current evidence suggests that periodontal treatment may contribute not only to improvements in clinical parameters but also to enhanced overall health perception and quality of life [29].

Smoking was also significantly associated with poorer OHRQoL in the bivariate analysis. The effect of tobacco use on periodontal progression has been extensively documented and is associated with vascular alterations, immune dysfunction, increased oxidative stress, and accelerated tissue destruction. However, its impact likely extends beyond biological mechanisms. Halitosis, esthetic alterations, functional impairment, and negative self-perception may additionally contribute to the psychosocial burden experienced by these patients. Previous studies have demonstrated that factors such as xerostomia, stress, and periodontal condition significantly influence perceptions of OHRQoL [30], confirming the multifactorial nature of the subjective experience of periodontal disease.

The sociodemographic variables evaluated in the present study also showed an important influence on OHRQoL. Participants with lower educational attainment and lower socioeconomic status presented significantly more unfavorable scores, suggesting that social determinants play a relevant role in the perception of oral health. Socioeconomic inequalities may contribute to barriers in accessing treatment, lower health literacy, delayed therapeutic intervention, and greater cumulative periodontal disease burden over time. In addition, psychosocial vulnerability may intensify the perception of symptoms and functional limitations associated with the disease [31,32]. These findings reinforce the need to address periodontitis from a comprehensive perspective that considers not only clinical variables but also social and contextual factors.

An unexpected finding was the association between divorced marital status and lower OHIP-14-PD scores compared with single status. This result should be interpreted with particular caution because marital status is a broad and culturally dependent sociodemographic category that does not adequately capture social support, household composition, relationship quality, economic circumstances, or the time elapsed since marital separation. None of these factors were measured in the present study. Moreover, the partially adjusted model did not include several relevant socioeconomic, behavioral, and psychosocial variables, increasing the possibility of residual confounding or a sample-specific association. Therefore, this finding should not be interpreted as indicating that divorce has a beneficial or protective effect on OHRQoL. Further studies with larger and more diverse samples and more detailed measures of social and family context are needed to determine whether this association is reproducible.

Male sex was associated with higher OHIP-14-PD scores in the partially adjusted multivariable model. However, this association should also be interpreted cautiously. No statistically significant difference according to sex was observed in the bivariate analysis, and the regression model did not include several potentially relevant confounders, including age, smoking status, educational level, and socioeconomic status. Therefore, the observed association may partly reflect residual confounding or characteristics specific to the study sample rather than a consistent sex-related effect. Because healthcare-seeking behavior, psychological adaptation, disease perception, and sex-specific differences in periodontal severity were not directly assessed, the mechanisms underlying this finding cannot be determined from the present data. Further studies specifically designed to investigate sex-related differences in OHRQoL are required before firm conclusions can be drawn.

Among the methodological strengths of the present study, the use of the OHIP-14-PD allowed the assessment of patient-reported functional, physical, psychological, and social impacts associated with periodontal disease. The instrument demonstrated excellent internal consistency in the study population. In addition, periodontal classification was performed using standardized clinical and radiographic criteria according to the 2017 World Workshop system, enhancing diagnostic reproducibility and comparability with contemporary studies. The exploratory multivariable analysis provided additional information on the associations between selected participant characteristics and OHRQoL. However, because the model did not include all potentially relevant confounders, its findings should be interpreted as partially adjusted associations rather than as evidence of independent effects.

From a clinical perspective, the findings suggest that periodontal staging may be complemented by patient-reported outcome measures, particularly in patients with advanced disease. Clinical and radiographic parameters describe periodontal tissue destruction, tooth loss, functional complexity, and treatment needs but may not fully reflect the pain, masticatory difficulties, psychological discomfort, social limitations, and concerns experienced by the patient. The markedly higher OHIP-14-PD scores observed among participants with stage IV periodontitis indicate a greater perceived oral health burden compared with stage II disease. Incorporating a brief OHRQoL questionnaire during the initial periodontal assessment and subsequent follow-up may help clinicians identify problems that are not evident from probing depth, clinical attachment loss, tooth loss, or radiographic findings alone. This information may support shared decision-making, improve communication regarding treatment expectations, and guide individualized priorities such as pain management, preservation or restoration of masticatory function, periodontal maintenance, smoking cessation counseling, and coordination of care for patients with diabetes. Patient-reported outcomes should not replace conventional clinical periodontal measures but may provide complementary information for evaluating treatment needs, patient concerns, and perceived treatment outcomes. Nevertheless, because of the cross-sectional design and the partially adjusted model, the present findings do not demonstrate that advanced periodontitis directly causes deterioration in OHRQoL.

Although the final sample exceeded the a priori requirement for the primary comparison between stage II and stage IV periodontitis, statistical adequacy for this specific comparison does not eliminate the limitations associated with the modest sample size and single-center recruitment. The study was not designed to provide population-level estimates, establish causal effects, or support broad generalization to patients receiving care in other clinical, geographic, or socioeconomic settings. Accordingly, the observed association should be regarded as sample-specific and hypothesis-generating until confirmed in larger multicenter longitudinal cohorts.

Several limitations should be considered when interpreting the results. First, the cross-sectional design precludes the establishment of causal or temporal relationships between periodontitis severity and deterioration in OHRQoL. Reverse causality also cannot be excluded, because poorer perceived oral health may influence oral hygiene behaviors, treatment adherence, and healthcare-seeking patterns, which may in turn affect periodontal status. Second, the use of a non-probability consecutive sample recruited from a single university periodontal clinic in Mexico limits the external validity of the findings. Patients seeking care at a university clinic may differ from individuals receiving care in private practices, community health services, or other geographic and socioeconomic settings in terms of disease severity, healthcare-seeking behavior, treatment accessibility, and systemic health characteristics. Therefore, the observed associations may not be directly generalizable to the broader Mexican population or to populations in other countries. Although consecutive recruitment reduced discretionary participant selection, selection bias related to the single-center clinical setting cannot be excluded.

Third, smoking status and diabetes mellitus were based on participant self-report and were not independently verified through medical records, biochemical testing, glycemic markers, or detailed smoking exposure measures. Consequently, recall error, social desirability bias, underreporting of smoking, undiagnosed diabetes, and exposure misclassification cannot be excluded. Moreover, the absence of information on smoking intensity and duration, pack-years, diabetes duration, and glycemic control may have resulted in residual confounding. Fourth, the exploratory multivariable model was only partially adjusted and did not include other potentially relevant confounders, such as age, smoking status, educational level, and socioeconomic status. Therefore, the regression findings should be interpreted cautiously, and residual confounding cannot be ruled out. The moderate explanatory capacity of the model (R^2^ = 0.38) also suggests that additional clinical, behavioral, socioeconomic, and psychosocial factors may account for a substantial proportion of the variability in OHRQoL.

Additionally, potentially relevant psychosocial variables, such as anxiety, depression, perceived stress, social support, and coping mechanisms, were not assessed and may influence subjective perceptions of quality of life. Other oral conditions that may affect OHRQoL, including dental caries, xerostomia, non-periodontal pain, non-periodontal tooth loss, masticatory dysfunction, and prosthetic status, were also not comprehensively evaluated. The inclusion of only stage II and stage IV periodontitis limits the generalizability of the findings to patients with stage I or stage III disease and precludes evaluation of a complete severity gradient across all periodontal stages. Future research should address the principal methodological weaknesses of the current evidence, including cross-sectional designs, clinic-based samples, restricted inclusion of periodontal stages, incomplete adjustment for confounding, reliance on self-reported behavioral and systemic variables, and assessment of OHRQoL at a single time point. Multicenter prospective studies should include patients across all periodontitis stages and grades, use repeated clinical and patient-reported measurements, and comprehensively assess smoking exposure, glycemic control, socioeconomic conditions, psychosocial factors, tooth loss, masticatory function, prosthetic status, and other oral diseases. Specific research questions should determine whether periodontal treatment produces clinically meaningful and sustained improvements in OHRQoL; whether tooth loss, masticatory dysfunction, pain, and psychological distress mediate the association between periodontitis severity and OHRQoL; and whether these relationships differ according to periodontitis stage and grade, diabetes control, smoking exposure, or socioeconomic context.

Overall, the findings indicate that patients with stage IV periodontitis reported a greater perceived oral health burden than those with stage II disease. This difference highlights the importance of considering both periodontal status and the patient’s subjective experience during clinical assessment. The integration of OHRQoL measures with conventional clinical and radiographic evaluation may contribute to more comprehensive and patient-centered periodontal care. However, these findings should be regarded as exploratory and interpreted in light of the cross-sectional design, the partially adjusted multivariable model, and the restricted inclusion of periodontal stages.

## 5. Conclusions

In this single-center cross-sectional clinical sample, patients with stage IV periodontitis reported poorer oral health-related quality of life than those with stage II periodontitis. The findings support an association between periodontal severity and OHIP-14-PD scores within the studied population but do not establish a causal effect of periodontitis severity on OHRQoL. Although the sample exceeded the prespecified requirement for the primary comparison, its modest size, single-center recruitment, restricted inclusion of periodontal stages, and partially adjusted analysis limit the precision and generalizability of the findings. Larger multicenter longitudinal studies are required to confirm this association and determine whether periodontal treatment results in clinically meaningful and sustained improvements in OHRQoL.

## Figures and Tables

**Table 1:** OHRQoL scores according to sociodemographic characteristics, risk factors, and periodontal status.

Variable	Category	*n* (%)	Mean ± SD	Median (IQR)	*p*-Value
Sex	Female	82 (60.3)	24.1 ± 14.4	19 (14–39)	0.326
	Male	54 (39.7)	26.3 ± 12.7	20 (16–38)	
Marital status	Single	17 (12.5%)	27.8 ± 13.2	25 (15–39)	<0.001
	Married	51 (37.5%)	22.4 ± 12.8	18 (13–31)	
	Divorced	50 (36.8%)	16.9 ± 10.4	14 (9–24)	
	Widowed	13 (10%)	34.6 ± 11.7	36 (25–45)	
Educational level	Primary education	45 (33.1)	31.5 ± 12.1	33 (20–41)	<0.001
	Secondary education	45 (33.1)	24.7 ± 12.8	21 (15–35)	
	Higher education	43 (31.6)	18.9 ± 11.4	16 (10–26)	
Socioeconomic status	Low	45 (33.1)	31.8 ± 13.0	34 (21–42)	<0.001
	High	91 (66.9)	21.6 ± 12.4	18 (12–30)	
Smoking status	No	78 (57.4)	21.2 ± 13.1	16 (14–30)	0.001
	Yes	58 (42.6)	29.7 ± 12.9	32 (17–41)	
Diabetes mellitus	No	68 (50.0)	20.6 ± 13.1	17 (12–29)	<0.001
	Yes	68 (50.0)	29.0 ± 12.9	27.5 (16–41)	
Periodontitis severity	Stage II	61 (44.9)	18.6 ± 12.6	16 (10–23)	<0.001
	Stage IV	75 (55.1)	30.1 ± 12.4	33 (17–41)	

Abbreviations: SD, standard deviation; IQR, interquartile range; OHRQoL, oral health-related quality of life.

**Table 2:** Exploratory partially adjusted multivariable linear regression analysis of factors associated with OHIP-14-PD scores.

Variable	β	95% CI	*p*-Value
Male sex	4.2	0.1–8.2	0.042
Diabetes	6.5	2.1–10.9	0.004
Divorced	−10.9	−17.7 to −4.2	0.002
Stage IV periodontitis	4.6	0.4–8.7	0.031

Note: The multivariable analysis included 128 complete cases. Eight participants were excluded because of missing marital status data. No missing observations were identified for sex, diabetes mellitus, periodontitis stage, or OHIP-14-PD scores, and no imputation was performed.

## Data Availability

The data to support the findings of this study will be available on request from the corresponding author.
